# Growth characteristics and therapeutic decision markers in von Hippel-Lindau disease patients with renal cell carcinoma

**DOI:** 10.1186/s13023-019-1206-2

**Published:** 2019-10-28

**Authors:** Patrick Schuhmacher, Emily Kim, Felix Hahn, Peggy Sekula, Cordula Annette Jilg, Christian Leiber, Hartmut P. Neumann, Wolfgang Schultze-Seemann, Gerd Walz, Stefan Zschiedrich

**Affiliations:** 1 Department of Nephrology and Primary Care, Medical Center – University of Freiburg, Faculty of Medicine, University of Freiburg, Hugstetter Str. 55, 79106 Freiburg, Germany; 2 Department of Radiation Oncology, Medical Center, Medical Center – University of Freiburg, Faculty of Medicine, University of Freiburg, Freiburg, Germany; 3 German Cancer Consortium (DKTK), Partner Site Freiburg, Freiburg, Germany; 4 Department of Diagnostic and Interventional Radiology, University Medical Center of the Johannes Gutenberg-University Mainz, Mainz, Germany; 5 Institute of Genetic Epidemiology, Medical Center, Medical Center – University of Freiburg, Faculty of Medicine, University of Freiburg, Freiburg, Germany; 6 Department of Urology, Medical Center, Medical Center – University of Freiburg, Faculty of Medicine, University of Freiburg, Freiburg, Germany

**Keywords:** Von Hippel-Lindau disease, VHL, Clear cell renal cell carcinoma, ccRCC, Growth characteristics, Therapeutic decision markers

## Abstract

**Background:**

Von Hippel-Lindau (VHL) disease is a multi-systemic hereditary disease associated with several benign and malignant tumor entities, including clear cell renal cell carcinoma (ccRCC). Since ccRCCs grow slowly, nephron sparing surgery is typically performed at a tumor diameter of 3–4 cm before the tumor metastasizes. However, in the case of recurrent disease, repeated surgical intervention can impair renal function. Therefore, it is crucial to optimize the timing for surgical interventions through a better understanding of the growth kinetics of ccRCCs in VHL. We investigated tumor growth kinetics and modern volumetric assessment to guide future therapeutic decisions.

**Results:**

The prevalence of ccRCC was 28% in a cohort of 510 VHL patients. Of 144 patients with ccRCC, 41 were followed with serial imaging which identified 102 renal tumors, which exhibited heterogeneous growth kinetics. ccRCCs grew at an average absolute growth rate of 0.287 cm/year, an average relative growth rate [*(lnV*_*1*_*-lnV*_*0*_*)/(t*_*1*_*-t*_*0*_*)*] of 0.42% and an average volume doubling time of 27.15 months. Women had a faster relative growth rate than men. Age and specific mutations did not influence tumor growth. Because of the tumor heterogeneity, we developed an additional cut-off volume of 40 cm^3^ for surgical intervention.

**Conclusions:**

Tumor heterogeneity and differences in growth kinetics is suggestive of a state of transient tumor dormancy in ccRCCs of VHL patients. The relative growth rate has not been previously described in other studies. Volumetric assessment as an additional parameter for surgical intervention could be a useful clinical tool and needs further investigation.

## Background

Von Hippel-Lindau syndrome is a rare autosomal-dominant syndrome with an incidence of 1/31,000–1/45,500 [1–3]. The syndrome is associated with multiple malignant and benign tumors such as hemangioblastoma of the central nervous system, hemangioma of the retina, endolymphatic sac tumors, epididymal and broad ligament cystadenoma, neuroendocrine pancreatic tumors, pheochromocytoma, and renal cell carcinoma.

The life expectancy of VHL patients can be limited by metastatic ccRCC and end stage renal failure due to repeated renal surgery [4]. To balance the risk of tumor metastasis and renal insufficiency due to repeated surgery, it is crucial to optimize the timing for nephron sparing surgery, which is the standard treatment. We routinely begin screening patients at age 10 with abdominal MRI, and continue surveillance every one to 2 years, depending on radiological findings, based on a center-specific regimen.

Several publications have tried to describe the natural growth kinetics of ccRCCs within VHL patients. However, results between these studies were highly discrepant, mostly likely due to the small sample size [5–7]. VHL guidelines recommend nephron sparing surgery at 3–4 cm tumor diameter, depending on the specific tumor location and contact to surrounding vessels [8–12]. Recurrent surgical intervention is limited by sequelae, including residual tissue scarring and damage of the surrounding organs or vessels. An optimal timing of surgical intervention is crucial for preserving kidney function. However, an in depth investigation of ccRCC growth characteristics with accurate sizing and kinetic information is lacking.

The University Medical Center of Freiburg is specialized in VHL patients, currently serving more than 500 patients. The present study investigated growth kinetics and prognostic factors by generating virtual 3D tumor models, and comparing the resulting volumetry to traditional calculations of tumor volume relying on three parameters of length, width, and depth.

## Results

### Patient characteristics

Patients presenting at our VHL center from 2001 to 2016 were retrospectively evaluated. Of 510 VHL patients, 144 patients were diagnosed with ccRCC (28%). Forty-one of the 144 RCC patients had a minimum of three consecutive abdominal MRIs. Patient characteristics are depicted in Table 1.

**Table 1:** Characteristics of the 41 observed patients

Characteristic	Absolute	Mean	Range	Median	Standard deviation (SD)
Total number of VHL patients	510	–	–	–	–
- VHL patients with RCC	144	–	–	–	–
- Minimum of three MRIs	41	–	–	–	–
Gender (n)					
- Male	17	–	–	–	–
- Female	24	–	–	–	–
Number of tumors	102	–	–	–	–
- Left kidney	41	–	1–3	–	–
- Right kidney	61	–	1–8	–	–
Mean age at initial RCC diagnosis	–	35.57	20–72	32	11.96
Tumor size at initial description in cm^3^	–	4.47	0.271–35.9	2.75	5.61
Tumor size at the end of observation cm^3^	–	19.74	0.6–88	13.4	17.88
Follow-up in months	–	52.21	18–149	43.8	26.93

Thirty-nine of 41 patients had a genetically confirmed *VHL* mutation; 2 of 41 had a clinical diagnosis of VHL. Twenty-four patients were female, 17 male. The 41 patients showed a total number of 102 tumors. The number of tumors per patient was between 1 and 10 tumors. Forty-one tumors were localized in the left kidney, 61 tumors in the right kidney. The average age of ccRCC diagnosis was 35.6 years (range 20–72 years). The average size of the tumors at initial observation was 4.47 cm^3^; the size at the end of the observation was 19.74 cm^3^. The mean follow-up time was 52.2 months with a range of 18–149 months (Table 1).

### Growth kinetics

**Fig. 1:**
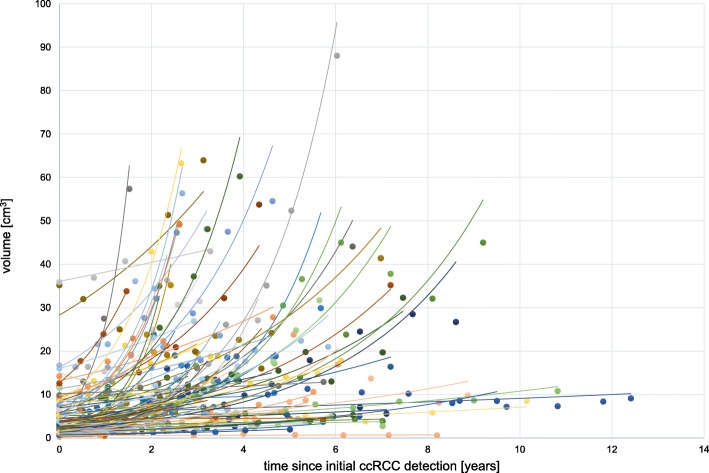
Growth curves of all 102 ccRCC since intial detection

**Fig. 2:**
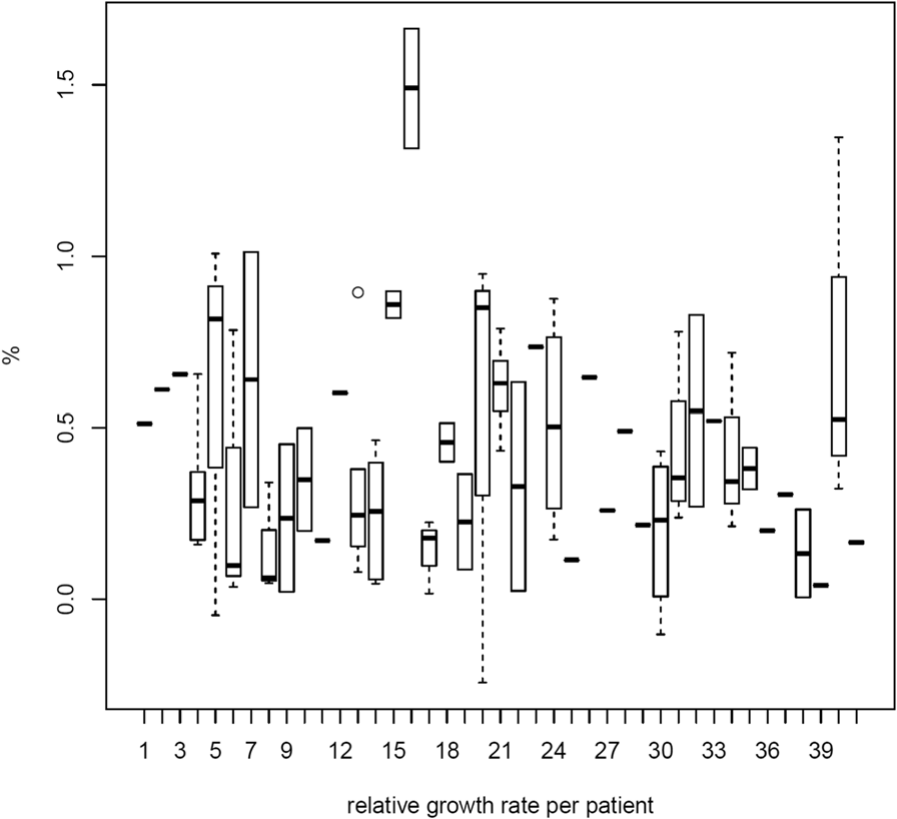
Boxplot graph of RGR per patient

**Fig. 3:**
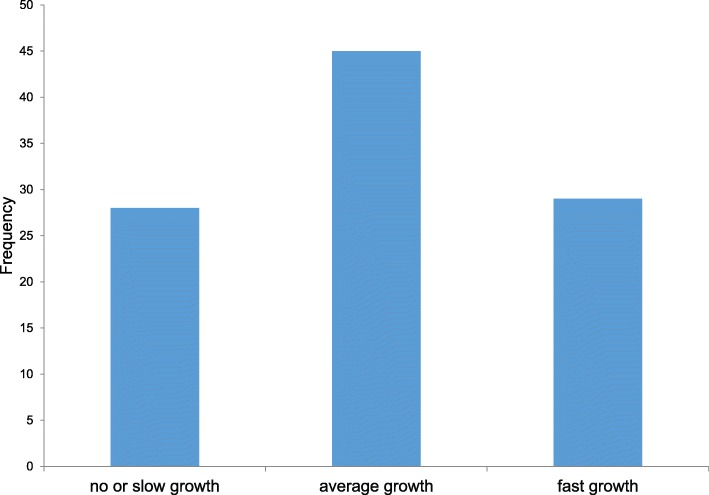
Classification of RGR into three subgroups; no or slow growth (RGR < 0.2%), average growth (0.2 - 0.6%) and fast growth (> 0.6%)

Figure 1 depicts the growth kinetics of the 102 analysed ccRCCs. The mean relative growth rate (RGR) was 0.42% per year, with a range of − 0.224-1.664%. The mean RGR for female patients and males patients was 0.48 and 0.35% per year, respectively. The average volume doubling time was 27.15 months. The absolute growth rate of the ccRCCs ranged from − 0.24-2.74 cm/year with a mean growth rate of 0.287 cm/year (Table 2). Heterogeneity of tumor growth rates was observed between the patients as well as the multiple tumors of individual patients (Fig. 2). We observed a large variance in the growth rates of tumors and formed three groups to describe the growth kinetics: 27.5% (28/102) had slow growth tumors (< 0.2%), 44.1% (45/102) moderate growth tumors (0.2–0.6%) and 28.4% (29/102) rapid growth tumors (> 0.6%) (Fig. 3). Negative growth was associated with cystic components of the tumor developing in follow-up which is explained by the fact that cysts may shrink or vanish.

**Table 2:** Growth kinetics of 102 ccRCC

Characteristic	Mean	Range	Median	Standard deviation (SD)
Relative growth rate	0.42	−0.244-1.664	0.35	0.33
- Female	0.48	−0.103-1.007	0.44	0.36
- Male	0.35	−0.244-1.664	0.34	0.27
Volume doubling time in months	27.15	− 671-309	21.83	87.62
Absolute growth rate in cm/year	0.287	−0.24-2.74	0.23	0.29

### Candidate prognostic factors of ccRCC growth

**Fig. 4:**
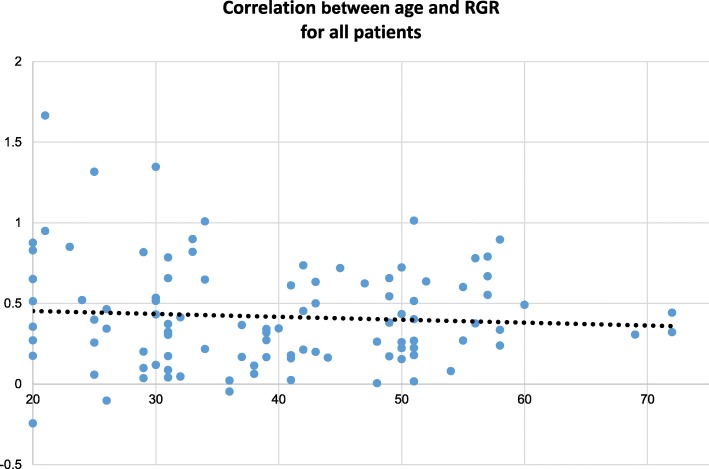
Correlation between age at first description of tumor and RGR; x-axis: age at first detection of tumor; y-axis: RGR in % per year

There was a significant difference in the RGR for men (Mean = 0.34; SD = 0.27) and women (Mean = 0.48; SD = 0.36), *p* = 0.03 using the t-test for unequal variances. When using a random intercept model, the observed difference in growth between male and female patient was not significant anymore (effect = − 0.14; SE = 0.08; *p* = 0.08, Table 3). There was no significant difference between the RGRs of the different germline *VHL* mutations and those of the entire cohort. Furthermore, there was no overall influence of age on the growth rate of all 102 tumors by Pearson correlation (Fig. 4). These results for the influence of age and the different mutations were confirmed in the random intercept model.

**Table 3:** Results of the linear random intercept model

Outcome: RGR			Result	
	Fixed effect	AIC	β (SE)	*P*-value
1	none	62.8	–	–
2	Sex (reference = female)	61.9		0.08
	- male		−0.14 (0.08)	
3	Age	64.3	−0.002 (0.003)	0.48
4	Mutation (reference = large deletion)	69.0		0.57
	- nonsense		0.05 (0.11)	
	- missense		0.24 (0.12)	
	- frameshift		0.05 (0.14)	
	- splice		0.09 (0.15)	
	- other		0.02 (0.10)	

Models 1 to 4 were separately fitted. Reported effects (SEs) relate to modeled fixed effect variable. *P*-values were obtained from χ^2^-test comparing the respective model to model 1. AIC, Akaike’s information criterion of model fit

### Establishing a volume as a cut-off for a surgical intervention

From 2001 to 2016 40 ccRCC were removed by nephron sparing surgery from 17 patients at our center. The timing of surgery was based on intervention at a maximum tumor diameter of 3–4 cm. Re-analyzing the tumor volume at the time of a surgical intervention revealed that the tumors were removed at a mean size of 41.3 cm^3^. The average age was 43.59 years at intervention. The mean number of interventions was 1.55 per patient.

### Dormant tumors and metastatic disease

**Fig. 5:**
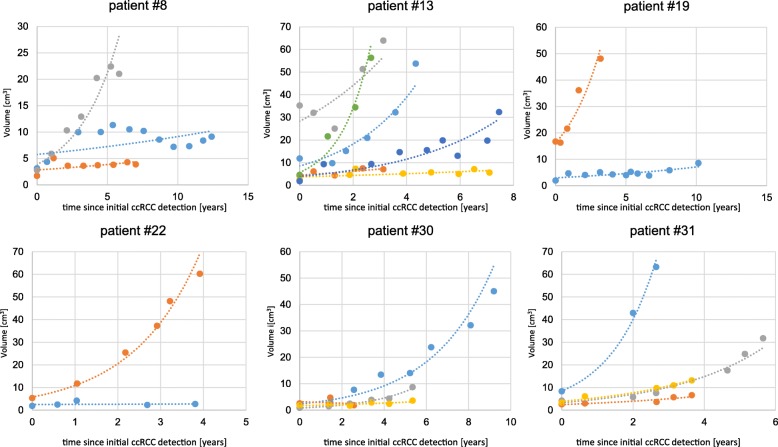
“Dormant tumors” next to proliferating tumors within one patient – tumor growth curves of 6 exemplary patients; x-axis: time since first description of tumor in years; y-axis: volume in cm^3^

Figure 5 depicts the growth kinetics of 6 exemplary patients with multiple ccRCCs. Within patients, tumors with rapid growth and tumors with slow growth exist next to each other. In the current observation, no patient had metastatic disease in follow-up.

## Discussion

The ccRCC incidence of 28% in our cohort falls in the lower range of previous reports (24–55.6%) [13–17]. This is the second largest cohort reported since Ong et al. with 573 patients and an incidence of 35% [13]; other reported cohorts are much smaller so one might expect the incidence to be around 30% in general.

At our center, the average age at first ccRCC diagnosis was 35.7 years. This relatively young age of first presentation is likely to result from the center screening protocol which enables tumors to be detected at an early stage. Other groups reported age at first ccRCC diagnosis between 36 and 39.7 years [13, 16–18]. The mean initial volume of ccRCC was smaller with 4.47 cm^3^ compared to former publications of our center with 7.2 cm^3^ [7]. This may be due to either an earlier detection of disease or a more accurate 3D reconstruction of ccRCC volume in our study.

Precise volumetric measurements, early detection and long follow-up of 15 years of our study may significantly contribute to the current discussion whether there is a difference in growth kinetics of ccRCC in VHL versus growth kinetics of sporadic ccRCC. The absolute growth rate of 0.287 cm/year of our VHL cohort is very much in line with sporadic ccRCC growth in a meta-analysis of Chawla et al. with 0.28 cm/year [19].

We found exponential tumor growth varied both inter-individually and intra-individually. The broad range of our absolute growth rate (− 0.24–2.74 cm/year) reflects similar results found before (0.04–2.2 cm/year) [5, 20]. Analyzing these results with a linear intercept model, there was no statistical significant influence factor as gender, age or type of germline *VHL* mutation. The few cases of tumor shrinkage were probably due to tumors with additional cystic lesions that could not be distinguished in early stage.

There was no case of metastatic ccRCC in the investigated cohort with a threshold to surgery of 4 cm maximum with a mean follow-up time of 52.2 months. Metastasis of a ccRCC in VHL with a 3–4 cm diameter has been reported; these authors suggested a 3 cm threshold for resection [4, 12]. However, Neumann et al. reported no metastatic disease up to 7 cm tumor diameter [21]. Jilg et al. demonstrated that careful observation of tumor growth up to 4 cm diameters can postpone a second intervention by 27.8 months in median [7].

## Conclusions

The findings of this study support a 4 cm cut-off for NSS. In the light of these heterogeneous study results, we feel that solely measuring diameter might be misleading. Measuring tumor volume by calculating the volume of an ellipsoid tends to under- or overestimate the volume due to the implied perfect shape of an ellipsoid in comparison to the real shape. A volumetric analysis with the *TeraRecon Aquarius iNtuition* or comparable programs should be recommended for therapeutic decision-making.

## Patients and methods

After institutional ethics board approval (EK 391/16), we retrospectively analysed patients diagnosed with ccRCC at our center. VHL disease was diagnosed by genetic testing and at least one typical tumor manifestation. In case of negative genetic testing of the *VHL* gene, VHL diagnosis was based on at least one hemangioblastoma of the retina or one hemangioblastoma in the central nervous system in combination with an additional manifestation (hemangioma of the retina, hemangioblastoma of the central nervous system, endolymphatic sac tumors, epididymal or broad ligament cystadenoma, neuroendocrine pancreatic tumors, pheochromocytoma or ccRCC).

**Fig. 6:**
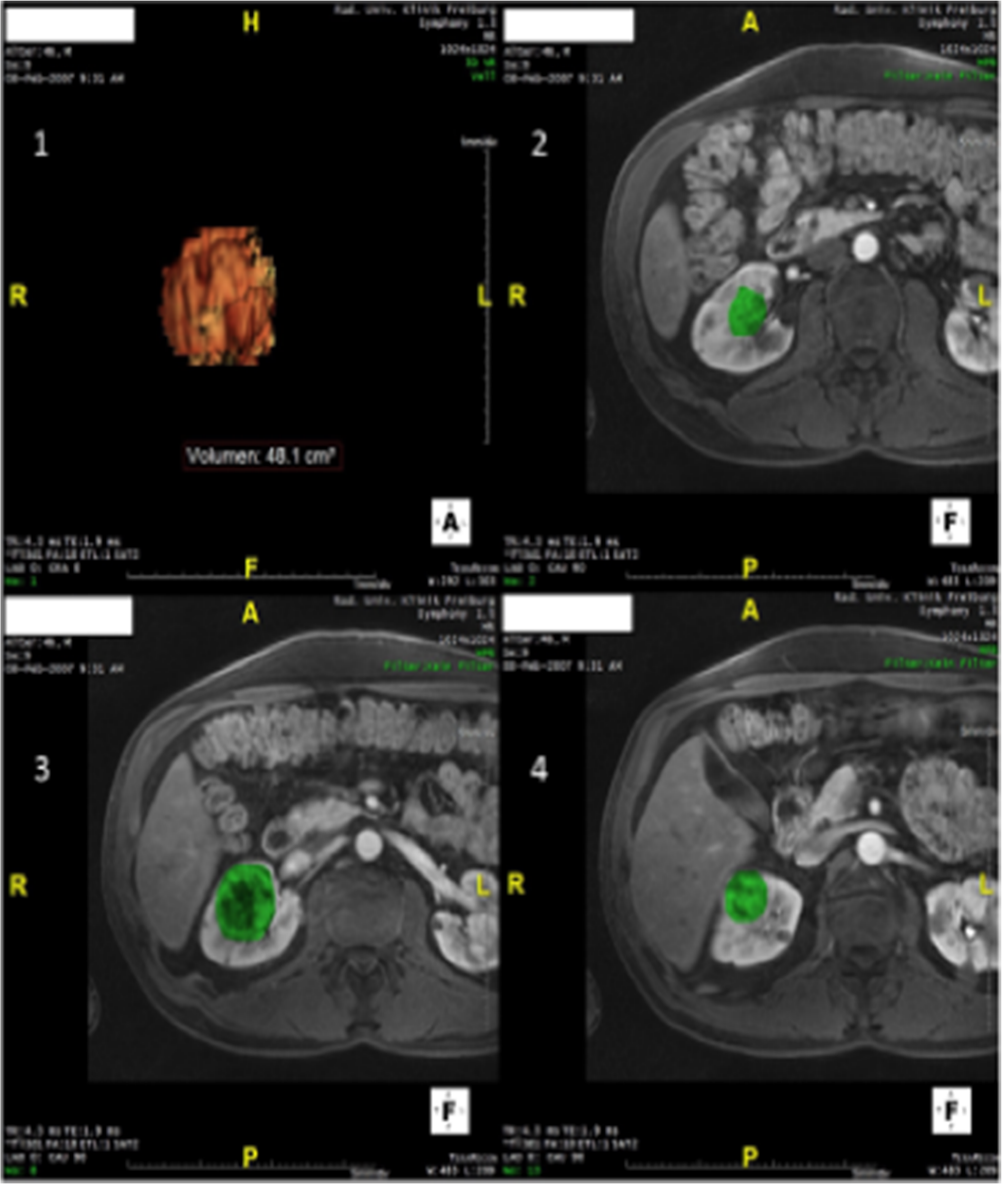
3D-reconstruction of a ccRCC (1); region of interest in different slices (2-4)

A total of 510 VHL patients were seen in the VHL clinic from January, 1st 2001 until January, 1st 2016 at the University Medical Center Freiburg. All patients with radiological detected ccRCC and a minimum of three consecutive magnetic resonance imagings performed with no greater gap than three years were included in this study. Selection of the ccRCCs was based on the documented findings of the radiology department of the University Medical Center Freiburg, extracted from the radiology information system. Image analysis was performed using *IMPAX EE R20 XIV*© diagnostic software. Routinely acquired contrast-enhanced thin-slice axial T1-weighted volumetric interpolated breath-hold examination (VIBE) sequences acquired at 1.5 Tesla Siemens scanners were used for volumetric analyses which were performed with *TeraRecon Aquarius iNtuition 4.4.12*© plug-in software (example: Fig. 6)*.*

To create an exponential growth curve for each tumor, a best fit model with the following formula was used:

$$ \mathrm{Y}={\mathrm{V}}_0\ast {\mathrm{e}}^{\mathrm{k}\ast \mathrm{x}} $$

The relative growth rate was calculated using the following formula:

$$ \mathrm{RGR}=\left({\mathrm{lnV}}_1-{\mathrm{lnV}}_0\right)/\left({\mathrm{t}}_1-{\mathrm{t}}_0\right) $$

The volume doubling time in months was calculated by the following equation:

$$ \mathrm{VDT}=\left({\mathrm{t}}_1-{\mathrm{t}}_0\right)\ast \log 2/{\mathrm{logV}}_1-\log\ {\mathrm{V}}_0 $$

V_1_=Volume at final MRI; V_0_= Volume at initial MRI; t_0_=time at initial MRI; t_1_=time at final MRI

To determine a linear correlation between two variables, Pearson’s correlation coefficient was calculated. Statistical significance of differences was evaluated with the t-test for either different variance (welsh test) and for same variance. In addition, a linear random intercept model was fitted to resolve non-independence due to patients having several tumors. The limit for statistical significance was defined as *p* < 0.05.

## Data Availability

The datasets generated and/or analyzed during the current study are not publicly available due to patient data safety restrictions but are available from the corresponding author on reasonable request.

## Abbreviations

ccRCC: Clear cell Renal Cell Carcinoma
SD: Standard deviation
VHL: Von Hippel-Lindau

## References

[1] Maher ER, Iselius L, Yates JR, Littler M, Benjamin C, Harris R (1991). Von Hippel-Lindau disease: a genetic study. J Med Genet.

[2] Neumann HP, Wiestler OD (1991). Clustering of features of von Hippel-Lindau syndrome: evidence for a complex genetic locus. Lancet (London, England).

[3] Maddock IR, Moran A, Maher ER, Teare MD, Norman A, Payne SJ (1996). A genetic register for von Hippel-Lindau disease. J Med Genet.

[4] Walther MM, Choyke PL, Glenn G, Lyne JC, Rayford W, Venzon D (1999). Renal cancer in families with hereditary renal cancer: prospective analysis of a tumor size threshold for renal parenchymal sparing surgery. J Urol.

[5] Hes FJ, Slootweg PJ, van Vroonhoven TJ, Hene RJ, Feldberg MA, Zewald RA (1999). Management of renal cell carcinoma in von Hippel-Lindau disease. Eur J Clin Investig.

[6] Ploussard G, Droupy S, Ferlicot S, Ples R, Rocher L, Richard S (2007). Local recurrence after nephron-sparing surgery in von Hippel-Lindau disease. Urology..

[7] Jilg CA, Neumann HP, Glasker S, Schafer O, Ardelt PU, Schwardt M (2012). Growth kinetics in von Hippel-Lindau-associated renal cell carcinoma. Urol Int.

[8] VHL Alliance. 2018 [Available from: http://www.vhl.org].

[9] Verein_VHL-betroffener_Familien_e.V. 2018 [Available from: http://www.hippel-lindau.de/].

[10] Binderup ML, Bisgaard ML, Harbud V, Moller HU, Gimsing S, Friis-Hansen L (2013). Von Hippel-Lindau disease (vHL). National clinical guideline for diagnosis and surveillance in Denmark. 3rd edition. Dan Med J.

[11] Jilg CA, Neumann HP, Glasker S, Schafer O, Leiber C, Ardelt PU (2012). Nephron sparing surgery in von Hippel-Lindau associated renal cell carcinoma; clinicopathological long-term follow-up. Familial Cancer.

[12] Duffey BG, Choyke PL, Glenn G, Grubb RL, Venzon D, Linehan WM (2004). The relationship between renal tumor size and metastases in patients with von Hippel-Lindau disease. J Urol.

[13] Ong KR, Woodward ER, Killick P, Lim C, Macdonald F, Maher ER (2007). Genotype-phenotype correlations in von Hippel-Lindau disease. Hum Mutat.

[14] Maher ER, Yates JR, Harries R, Benjamin C, Harris R, Moore AT (1990). Clinical features and natural history of von Hippel-Lindau disease. Q J Med.

[15] Goldfarb DA, Neumann HP, Penn I, Novick AC (1997). Results of renal transplantation in patients with renal cell carcinoma and von Hippel-Lindau disease. Transplantation..

[16] Kim WT, Ham WS, Ju HJ, Lee JS, Lee JS, Choi YD (2009). Clinical characteristics of renal cell carcinoma in Korean patients with von Hippel-Lindau disease compared to sporadic bilateral or multifocal renal cell carcinoma. J Korean Med Sci.

[17] Kwon T, Jeong IG, Pak S, You D, Song C, Hong JH (2014). Renal tumor size is an independent prognostic factor for overall survival in von Hippel-Lindau disease. J Cancer Res Clin Oncol.

[18] Steinbach F, Novick AC, Zincke H, Miller DP, Williams RD, Lund G (1995). Treatment of renal c cell carcinoma in von Hippel-Lindau disease: a multicenter study. J Urol.

[19] Chawla SN, Crispen PL, Hanlon AL, Greenberg RE, Chen DY, Uzzo RG (2006). The natural history of observed enhancing renal masses: meta-analysis and review of the world literature. J Urol.

[20] Choyke PL, Glenn GM, Walther MM, Zbar B, Weiss GH, Alexander RB (1992). The natural history of renal lesions in von Hippel-Lindau disease: a serial CT study in 28 patients. AJR Am J Roentgenol.

[21] Neumann HP, Bender BU, Berger DP, Laubenberger J, Schultze-Seemann W, Wetterauer U (1998). Prevalence, morphology and biology of renal cell carcinoma in von Hippel-Lindau disease compared to sporadic renal cell carcinoma. J Urol.

